# Topics of Public Interest

**Published:** 1918-06

**Authors:** 


### TOPICS OF PUBLIC INTEREST

**State Board Examinations, 1917.** 2605 graduates of 1917 and 4253 altogether, were examined. 96 colleges were represented. The failures were 7% for Class A colleges; 18.4% for B; 35.4% for C; 41.7% for foreign schools; 46.8% for undergraduates. 27-37 states refuse to examine graduates of the various Class C colleges. The maximum number of licenses was granted in 1906, the minimum in 1017—7865 and 4061 respectively. 38 states require 1 year of collegiate study, 30 two or more.

**The National Board of Medical Examiners** is recognized by Col., Del., Idaho, Ky., Md., N. H., N. C., N. D., Pa., R. I., Vt.

**Retired Medical Officers** of the Army, Navy and Public Health Service, are licensed without examination by Ala., Cal., Col., Ill., N. D., Va., Wis., and Porto Rico. (Note: It is none too early to consider the needs both of the civil population and of the medical profession after the war. Every man who has made good in the military and cognate services during the war is competent to practice his profession anywhere, quite aside from the natural tendency to reward his patriotism. This statement is purposely made in broad terms to include not only the medical but any other profession for which license is required or may be required by states. The medical profession, being the one mainly interested, should take action to secure legislation on this point.)

**An Imperative Appeal for Medical Officers.** An urgent and imperative appeal has just been issued by the Surgeon General of the United States Army, for doctors for the medical Reserve Corps.

There are today, 15,174 officers of the Medical Reserve Corps on active duty and the Medical Department has reached the limit of medical officers at the present time available for assignment. With these facts before the medical profession of this country, we believe that every doctor who is physically qualified for service between the age of 21 and 55 years, will come forward now and apply for a commission in the Medical Reserve Corps.

Drafts of men will continually follow drafts, each of which will require its proportionate number of medical officers and there are at this time on the available list of the Medical Reserve Corps, an insufficient number to meet the demands of these drafts.

**The Fifth Health Center** for Buffalo has been opened at Court and Franklin Sts. This dispensary is intended to serve the down-town section, including the water front, and is at the free disposal of persons living in that section who are without funds. Dr. Chas. Leone, a city physician, is in charge. Clinics will be held there regularly.

---

**Standard Names and Terms** proposed to be employed in military surgical work. The Medical Section of the Council of Defense is working to this end.

---

**Parcel-Post Mail** and fourth class mail sent to the U. S. Expeditionary Forces abroad or U. S. naval vessels need have no internal-revenue stamp affixed.

---

**Bureau of War-Risk Insurance** of the Treasury Department announces that in the six months since Oct. 6, 1917, 14 billions of insurance has been written. This was taken by approximately 1,700,000 soldiers, sailors and nurses. The average amount applied for was close to $8,500, the minimum permitted by the law being $1,000 and the maximum $10,000.

---

**The Minn. State Pharmaceutical Assn.**, at its annual convention and the Phila. Drug Exchange have gone on record in favor of requesting the Committees of Revision of the U. S. P. and N. F. to consider substitute formulas as a preparedness measure for the duration of the war.

---

**The Surg.-General** authorizes the following statement: 113 sick and wounded soldiers returned from the Am. Expeditionary Forces, were landed in the U. S. during the week ending April 26.

---

**Mrs. Charlotte Davenport**, who is 95 years old, is touring the country lecturing on the behalf of physical culture to the children in the schools. She has five sons and eighteen grand-sons in the French Army.

---

**The National War Work Council** of the Y. M. C. A. announces that the average American soldier in France does not spend more than 20 cents a day. That this modest sum covers pay for toilet articles, tobacco and sweets. That the men are all saving money and investing it in Liberty Bonds, War Saving Stamps and sending it home to their people.

---

**Some Paradoxes of the War.** It was prophecied that the development of long range, accurate aim and rapid fire, would necessitate action at long distance. No war has been fought with such small average distance between combatants. The conditions of trench warfare and the impossibility of burying the dead and of applying sanitary methods would theoretically produce so great disease incidence that military casualties would sink into insignificance. On the contrary, all of the armies of medically enlightened nations not only claim death rates from disease far below those formerly prevalent but even below those of the same age and sex group in conditions of peace. It was expected that high velocity missiles from small arms would produce comparatively insignificant wounds unless a vital spot were directly hit. On the contrary, these wounds have proved especially destructive. But, again, the actual recoveries, especially the return of 80-90% of the wounded to military service, is unprecedentedly high. Trench warfare should theoretically cause the majority of wounds to involve the head and upper extremity. Actually, leg injuries are 2-3 times as frequent as those of the arms and injuries below the waist 7 times as frequent as those above. The non-combatant troops of the medical department have the highest mortality and aviation officers are said to have the lowest relative mortality of all officers.

---

**Military Road.** N. Y. State has appropriated a million dollars to improve missing links between N. Y. City and Albany, Buffalo and the Pennsylvania line and to keep the road in good condition for war traffic. It may be remembered that we advocated this policy two years ago, long before the emergency occurred.

---

**The Medical Reserve Corps.** The recent German drive has hurried the transportation of troops to Europe and the army actually raised and in service, here or abroad, requires just about the number of medical men who have volunteered, there being a comparatively few who have accepted commissions and who have not been called to service. The Surgeon General's office is too much engaged with more practical duties to furnish either a list or statistics of the volunteers from this or any other section of the country, although we would be glad to publish such information. But it is immaterial whether Buffalo and western N. Y. have furnished more or less than their proportionate number. Such sacrifice to the country's needs, like Liberty loan allotments can be determined only approximately on the basis of population. So far as can be determined by inquiry, our territory has done its share on a mathematic basis. On the other hand, the opinion is generally expressed that this region has not been depleted of physicians to the degree either of suffering on the part of the laity or undue prosperity of the members of the profession left. The moral is plain, especially as some cities, especially in the south, are reported to have reached this condition. The age limits are 22-55, the pay liberal for young men, adequate for older men who have been successful and who have presumably been able to provide for their families. In plain terms of money, a reserve officer gets $2,000-3,000 according to the three ranks conferred, plus some perquisites which are liable to be restored to those pertaining in peace for the regular army or even increased beyond this point and thus equivalent to salaries 10% higher, with another 10% for foreign service automatically. He can get along comfortably on $1,000-1,200 for himself. Generally speaking, so far as can be determined by observation of groups of medical officers at training camps or in active duty, they are about equally divided into the three age groups 22-35, 35-45 and from 45 upward. Except that the reduction of graduates in the last ten years has left the medical profession top-heavy in age as compared with the population generally or any vocation or other group subject to a steady process of dying off at the top and growth at the bottom, this means an enormously greater proportionate volunteering of the older men. To allay the apprehensions of those patriotically inclined it may be said that, as a rule, the older men are perfectly able to withstand the physical strain and to adapt themselves to altered conditions of life and habit, especially to conform to discipline. The older one is, the more does varied experience count rather than actual length of life, even if that showed any tendency to being shortened by military service, the less does he have to think of his financial future, the more does he need a complete change, the less he has to sacrifice. Forget statistics and ultimate selfish considerations and decide the matter solely according to whether you can or cannot leave your private life.

---

**Chicago's Municipal Garbage Reduction Plant.** The grease and other material conserved was sold for $3,575 in Feb. and $6,236 in Mch. Buffalo has a most interesting plant of this kind which, we understand pays even greater actual amounts.

---

**Housing Capacity of Buffalo.** A recent census by the Health Dept. gives a total of 64,625 living places, divided as follows: 1-family houses 33,175; 2-family 25,847; tenement houses 4,309; rooming houses 1,075; lodging houses 50; boarding houses 94; hotels 76. Counting tenements as averaging 6 families, rooming, lodging and boarding houses as averaging 2 families and considering that hotels are for transients, this really means 113,161 dwelling units and corresponds quite closely to the accepted average of a trifle less than 5 persons to a family.

---

**Foreign Born Population of American Cities.** By different methods of calculation, we have estimated that the population of the U. S. is 45-55% foreign, in the sense of having reached this country since 1820, and, owing to the comparatively small immigration for the latter part of the colonial period and the beginning of the national period, the percentage would be only slightly larger if 1700 were taken as the dividing line. The south is more largely originally American than the north and the country districts than cities. Some have estimated that the population of few northern cities of large size is more than 10% colonial American. The percentages of literally foreign-born (German-born following), is stated as follows: St. Louis 18%, 6.95%; Detroit, 33%, 9.59%; Cleveland, 34%, 7.38%; Boston, 35%, —; Chicago, 35%, 8.34%; Bridgeport, 36%, —; New York, 41%, —; Cincinnati, —, 7.81%; Buffalo, —, 10.34%; Milwaukee, —, 17.33%.

---

**Commutation of Quarters for Officers.** In peace, officers are allowed quarters beginning with two rooms for a second lieutenant and increasing one room for each advanced grade. If quarters are not available commutation is allowed at the rate of S. QW. per room. In war, officers below the rank of major are allowed half a tent, majors a whole tent, or a small room in barracks of some sort in most of the present cantonments. A recent bill, signed by the president, restores the commutation allowance for officers actually having families for which they are providing quarters, in addition to an allowance for heat and light according to season. Just how the latter will be arranged, has not yet been announced but it will probably be in accordance with army regulations. This means for the Medical Reserve, that 1st lieutenants will have an addition to their pay of $36, captains $48, and majors $60 a month, which makes the service still more attractive.

---

**Telephone Rates.** For several years, the Bell Companies have been arguing as to the uneconomic features of dual systems. Having, in most places succeeded in killing competition, they now propose to raise the rates to something like the higher prices prevailing before the uneconomic dual system sprung up. There is only one answer to this sort of logic, strict government control and it has already been seriously suggested that both telegraph and telephone systems should be made part of the postal system as in most foreign countries. The postal service, having been originally and constitutionally reserved as a function of the federal government, methods of transmitting messages not discovered at the time, are legitimately included with written messages. If this were denied, it would be just as proper to hold that while letters written with a pen were mail matter, those written on a typewriter are not. Government control of telegraphs and telephones has worked well in most foreign countries, the rates being with few exceptions much lower than in the U. S. the exceptions being due to the use of the government monopoly as a substitute for other means of raising state funds.

---

**German Statistics.** It is of some interest to consider some of the recent statistics. Gen. Schultze, for example, has recently stated to the Reichstag that 629,000 men had been discharged for disability and that 750,000 sick and wounded had been returned to the service. It is usually considered that the wear and tear of an army, aside from military casualties is about equal to the increments from each annual class—i.e. about 1% of the population per annum. That is to say, Germany would lose about 700,000 men from her army each year simply from aging and general physical infirmity and would gain as many from the rising generation. The sickness and disability of our own army in this country, according to weekly reports, corresponds approximately to the incapacity of every man for some period, though there has been no terrible epidemic and the health has been good on the whole. Of course, such a statement includes venereal disease incidence, many minor complaints and really means that every thousand men yield only a trifle over a thousand days' incapacity for duty. Germany has definitely claimed that 90% of the wounded were returned to duty and the average proportion of recoveries should hold good for sickness. A few diseases, it is true, have very high mortality rates and those comparable with pneumonia and typhoid have mortality rates of somewhere between 20 and 10%, but diseases of such severity or greater, are comparatively rare while many, such as the exanthemata have quite low mortality and permanent disability rates. We can, therefore, scarcely reconcile the statement that the sick and wounded returned to the army are only about a fifth greater than the discharges to civil life. Cripples are stated in different places as numbering 70,000 discharged from service and 98,000 reckoned with altogether. Elsewhere, the total losses for the war are given as 2,000,000. If this statement means the dead, or even the dead plus 644,104 elsewhere admitted as captured (about 512,000) and missing (132,000), the number is out of proportion to any conceivable ratio of killed to total wounded, or to the number of cripples given. And, if the 2,000,000 means total definitive losses, it does not check up with the few rather doubtful numbers that may be taken as known quantities in an equation. In short, we cannot make the various figures harmonize with any previous experience in military losses of various kinds. Other statistics, partly based on English compilations of German official publications of casualties, partly on number and known size of divisions engaged on various fronts, partly on prisoners known to be in the various entente ally countries, partly on the balance of casualties between opponents, agree quite accurately on a system of cross checking, that the definitive German losses up to the end of 3½ years of war were 3½ million, with an estimate that the recent six weeks' heavy fighting has resulted in a total of about ½ million more, half of which would be returned to service after recovery from wounds. Generally speaking, the total definitive losses will be just about double the actual fatalities. We have previously alluded to the military axiom that a country cannot maintain more than 10% of its population in actual military service and that it cannot make good during an active and comparatively short war for losses beyond the non-military wear and tear, although it may temporarily withdraw men from industries, or draw on the feeble, aged or immature. The conclusion seems to be justified that Germany is down to about 3½ million men, including the ultimate returns to duty from the recent casualty list. Measurement of any conceivable western front shows that comparatively few reserves are available at an allowance of 5,000 men per mile, even if no account is taken of the needs on an eastern front or reinforcement of the Austro-Italian front. It is also obvious that when the man-power has been reduced to the minimum necessary to hold a given front, temporary disabilities count as powerfully as definitive, more so if we consider the man-power required to take care of the wounded. We prefer, however, to leave prophecies of duration of the war to others, taking these premises for what they are worth and with due allowance for errors in data.

---

**43 Women Physicians** have been sent into foreign medical service by the American Red Cross. They have been sent as individuals not as a unit. Dr. Regina Flood Keyes of Buffalo, Dr. Frances M. Flood of Elmira and Dr. Esther Parker of Ithaca are among the list.

---

**New Postage Stamp** for letters via Aeroplane is red, white and blue, showing an aeroplane and the denomination 24 cents.

---

**Mrs. Hanna G. Leslie** of Jamestown died April 8, age 100 years and 3 months.

---

**Right of French Soldier to Refuse Operation.** We have previously noted that the right of the American soldier in peace to refuse a major operation involving general anaesthesia of appreciable risk of death has been suspended during the war. Considering that the healthy soldier is compelled, in war, to incur a very appreciable risk of death, it is held that a board of surgeons may compel submission to any operation that will with reasonable probability, restore a disabled soldier to duty. This is entirely proper and we are somewhat surprised to note that a recent decision of the French Minister of War holds that a wounded soldier may refuse operation but that such refusal may be officially used to reduce future pensions to the basis of the probable net disability in case operation had been done. A recruit may also refuse an operation to remove disability existing before his mobilization.

---

**The Cartilage Co. was Indicted for Fraudulent** use of the mails and trial was begun before Judge Hazel in Buffalo, April 15. The testimony of expert witnesses as to the possibility of increasing stature by a stretching process was contradictory, one of the strongest points for the defense being a letter from Dr. Wm. L. Anderson, head of the Yale Physical Dept., to K. Leo Mingis, one of the defendants, to the effect that the latter might be on the right track. The case went to the jury May 3 and a disagreement was reported May 4.

---

**Schick Test in Diphtheria.** The existence of a considerable degree of hereditary immunity is a fact often overlooked. About 80% of the newborn give negative Schick reaction; at the age of 5, the proportion is reduced to about 50% while 80-90% of adults react negatively. Remembering that a negative Schick test means actual immunity, it will be seen that these percentages correspond well with the incidence of the disease at different ages.

**Anthrax** was reported early in May near Corning, two cows having died on the farm of Seldon Gridley of Caton. The rest of the herd was vaccinated.

